# Rethinking scale in AI-driven genomic medicine – The role of small biobanks

**DOI:** 10.3389/fgene.2026.1903325

**Published:** 2026-06-30

**Authors:** Laura Grech, Nikolai Paul Pace

**Affiliations:** 1 Department of Applied Biomedical Sciences, Faculty of Health Sciences, University of Malta, Msida, Malta; 2 Centre for Molecular Medicine and Biobanking, University of Malta, Msida, Malta; 3 Department of Anatomy, Faculty of Medicine and Surgery, University of Malta, Msida, Malta

**Keywords:** artificail intelligence (AI), biobanks, ethical, genomic medicine, legal and social issues (ELSI)

## Abstract

Artificial intelligence is rapidly advancing genomic medicine, but its clinical trustworthiness cannot be secured by larger datasets alone. This perspective argues that small biobanks, including national, regional, hospital-linked and disease-focused collections provide essential stress tests for AI-driven genomic medicine because they expose failures in population calibration, rare-variant interpretation, phenotype realism, privacy protection, and governance. Rather than serving primarily as substrates for training general-purpose models, small biobanks are most valuable as environments for external validation, local calibration, interpretability, federated analysis, and accountable deployment. Their local representativeness, clinical linkage, and governance structures can help determine whether AI predictions remain valid and clinically useful outside the large datasets on which they were developed. Trustworthy AI-powered genomic medicine will therefore depend not only on larger models and larger datasets, but also on smaller, well-governed biobanks that force those models to prove their validity in real-world settings.

## 1 Introduction

Artificial intelligence is rapidly being incorporated into genomic medicine. Deep learning now supports variant calling, splice site and missense variant interpretation, regulatory sequence modelling, phenotype matching, multi-omics integration, and prediction of molecular alterations from histology, radiology, and electronic health records (Duong and Solomon, 2025; Poplin et al., 2018; Cheng et al., 2023; Frazer et al., 2021; Jaganathan et al., 2019; Avsec et al., 2021). These developments are technically impressive and clinically relevant. They promise faster diagnosis, improved variant prioritisation, better molecular stratification, and more equitable access to genomic expertise. Yet the field risks adopting a misleading assumption: that the main route to trustworthy AI in genomic medicine is simply larger datasets.

## 2 Beyond scale: what small biobanks uniquely contribute

Throughout this article, ‘small biobanks’ refers to genomic collections whose scientific value derives from focus and context rather than scale. In practical terms they typically range from a few hundred to a few tens of thousands of participants - in contrast to mega-biobanks of several hundred thousand to millions - although this boundary is relative rather than absolute. It also depends on the disease, population, and analytical question at hand. The category spans several overlapping types: national biobanks of small or geographically isolated countries; regional or hospital-based collections linked to local clinical services; founder-population cohorts with distinctive allelic architecture and elevated rare-variant frequencies; and disease-focused collections assembled around a specific condition or phenotype. What unites them is not a fixed sample-size threshold but a shared reliance on local representativeness, robust governance, clinical linkage, population specificity, or focused disease ascertainment.

Scale matters, but scale is different from validity. Large biobanks provide statistical power, enable model training across many phenotypes, and provide the substrate for foundation-model approaches. However, many of the most important clinical failures of AI-driven genomic medicine emerge not in the average individual represented in a large training set, but in the populations, health systems, and data environments that differ from it. Small biobanks should thus not be viewed simply as underpowered versions of large-scale resources. In this context, ‘small’ refers not only to sample size, but to a different source of scientific value. These biobanks often derive their strength from local representativeness, robust governance, clinical linkage, population specificity, or focused disease ascertainment rather than from scale alone. When properly designed, they can therefore function as essential stress tests for genomic AI, revealing whether models remain valid, interpretable, and clinically useful outside the large datasets on which they were developed (Table 1). This commentary does not argue that small biobanks should replace large-scale resources for training general-purpose genomic AI models. Rather, it argues that their principal value lies downstream and alongside model development: in external validation, local calibration, interpretability, privacy engineering, governance design, and real-world deployment testing. These contributions are not unconditional, as small biobanks also carry real limitations–including reduced statistical power, phenotype sparsity, relatedness structure, and local ascertainment bias. We weigh these limitations alongside their strengths throughout the discussion that follows.

**TABLE 1:** Unique contributions of small biobanks to AI-driven genomic medicine, their associated limitations, and recommended mitigation strategies.

Dimension	Unique contribution of small biobanks	Associated limitation	Recommended mitigation strategy
Population calibration	Expose reduced portability of models (e.g., PRS) trained on large, ancestrally narrow cohorts by testing them in locally representative populations	Reduced statistical power; relatedness structure and local ascertainment bias can distort estimates	External validation in deployment populations; multi-ancestry training and local recalibration; explicit reporting of relatedness and ascertainment
Rare-variant interpretation	Contain founder alleles, enriched low-frequency variants, and population-specific haplotypes that challenge generic predictors	Risk of false reassurance or false alarm when generic predictions are applied without local context	Treat AI tools as calibrated evidence generators, not autonomous classifiers; integrate local allele frequencies, segregation, and ACMG/AMP evidence
Phenotype realism	Linkage to local clinical workflows reveals whether models learn biology versus data-entry convention or service structure	Phenotype sparsity; noisy, temporally unstable labels shaped by coding and health-system behaviour	Document phenotype provenance (source, coding system, certainty); test for confounding in image- and EHR-derived predictions
Privacy	In small populations, combinations of variants, geography, phenotype, and demographics become highly distinctive, exposing re-identification risks that large cohorts mask	Greater vulnerability to membership-inference and reconstruction attacks via beacons and aggregate queries	Engineer AI-ready access: tiered access, audit trails, query-rate limits, rare-variant suppression, output checking, controlled APIs, governance over model export
Infrastructure and governance	Serve as governance testbeds for computable, traceable, reusable data and evolving consent models	Most small biobanks lack legal/technical infrastructure; dynamic consent adds administrative burden and consent fatigue	Harmonised metadata and versioned pipelines; FAIR-but-controlled access; tiered/dynamic consent where feasible; model cards, data sheets, and audit logs
Biological interpretability	Link variation to rich local clinical data, family history, and founder effects to test whether predictions reflect real mechanisms	Limited representation of rare outcomes; overfitting risk in small samples	Use as hypothesis-generation and mechanism-testing environments via segregation analysis, functional assays, and multi-omics validation

## 3 Stress-testing genomic AI in real-world populations

The first stress test is population calibration. Genomic AI models learn from the distribution of variation, linkage disequilibrium, phenotype definitions, technical artefacts, and ascertainment structures present in their training data (Gündüz et al., 2024; Novakovsky et al., 2023). These distributions are not universal. Polygenic risk scores provide the clearest warning: models derived predominantly from European-ancestry cohorts, and often from a limited subset of European populations, can show reduced portability across genetically diverse populations (Lennon et al., 2024; Moreno-Grau et al., 2024). Multi-ancestry training, local calibration, and responsible use of population descriptors can improve performance, but they do not remove the need for external validation in the populations where tools will be deployed (Tsuo et al., 2025; Smith et al., 2025). Small biobanks can be valuable precisely because they have the potential to expose these issues. A model that performs well in a large reference dataset but fails in a smaller, locally representative cohort is not clinically robust (Carress et al., 2021; Huffman, 2018).

The second stress test is rare-variant interpretation. AI-based predictors such as AlphaMissense, EVE, SpliceAI, and sequence-to-expression models have changed what is technically possible in variant prioritisation (Cheng et al., 2023; Frazer et al., 2021; Jaganathan et al., 2019; Avsec et al., 2021). They can rank millions of variants, highlight likely damaging substitutions, identify cryptic splice effects, and infer regulatory impact. However, clinical interpretation is not a ranking problem alone. It is an evidentiary process that must integrate population frequency, inheritance, segregation, phenotype specificity, disease mechanism, functional data, and gene-disease validity (Richards et al., 2015; Pejave et al., 2022; Walker et al., 2023; McEwen et al., 2026). These tools should therefore be treated as calibrated evidence generators, not as autonomous clinical classifiers. Small biobanks often contain the allelic architectures that challenge generic predictors: founder alleles, enriched low-frequency variants, cryptic relatedness, population-specific haplotypes, and local ascertainment patterns. These features can generate false reassurance or false alarm when AI predictions are incorporated into clinical interpretation without local calibration.

The third stress test is phenotype realism. AI genomic medicine increasingly aims to integrate molecular data with clinical records, imaging, pathology, laboratory data, prescriptions, and longitudinal outcomes. In principle, this multi-modal approach is powerful. In practice, clinical phenotypes are noisy, temporally unstable, and shaped by health-system behaviour. Diagnostic codes may reflect billing practices, referral pathways, diagnostic access, or clinical fashion rather than biology (Biggin et al., 2023; Marshall et al., 2022). These are domain-shift problems, where the statistical relationship between inputs, labels, and outcomes changes when a model moves from one institution, population, assay platform, or clinical workflow to another. Small biobanks linked to local clinical workflows can reveal whether a model is learning biology, data-entry convention, or service structure. This is particularly important when AI predicts genomic or molecular alterations from digital pathology or radiological images, or EHR-derived phenotypes. Apparent biological signal may be confounded by staining protocols, scanner type, hospital site or treatment access.

The fourth stress test is privacy. In the EU, genetic data are explicitly regulated as a special category of personal data under the GDPR. In the United States, genetic information is protected through a more fragmented framework, including HIPAA when the information is individually identifiable and held by covered entities, and GINA in specific anti-discrimination contexts. Across both jurisdictions, the regulatory concern rests on two linked features: genetic data can contribute to individual identification and can reveal sensitive information about individuals and, in some cases, their biological relatives (Rahnasto, 2023). In small populations, privacy risks are amplified because combinations of variants, geographic origin, phenotype, and demographic metadata can become highly distinctive. Genomic beacons and aggregate query systems were designed to facilitate data discovery, but the literature on membership inference and genome reconstruction attacks shows that apparently limited query access can still carry risk (Saleem et al., 2025; Thomas et al., 2024). This does not mean that small biobanks should retreat from data sharing. It means that AI-ready access must be deliberately engineered: tiered access, audit trails, query-rate controls, rare-variant suppression where appropriate, output checking, controlled APIs, data-use agreements, and governance over model export as well as data export. In practice, these controls can map onto a tiered model: open aggregate statistics, registered access for credentialed researchers, and controlled access via a data access committee for individual-level data. Interoperable standards such as the GA4GH framework, the Passport and Authentication and Authorization Infrastructure, and the Data Use Ontology allow access conditions to be expressed in machine-readable form, while access-aware discovery protocols such as Beacon v2 enable queries that limit membership-inference risk (Rehm et al., 2021; Voisin et al., 2021; Lawson et al., 2021; Rambla et al., 2022).

The fifth stress test is infrastructure. A biobank is not AI-ready because it has genome data. It becomes AI-ready when its data is computable, traceable, governed, and reusable. This requires harmonised metadata, versioned variant-calling pipelines, reference-genome documentation, phenotype provenance, ancestry descriptors, consent status, data quality metrics, and clear rules for secondary use. FAIR data portals are becoming central to genomic science, but FAIR must not be mistaken for unrestricted openness (Speir et al., 2025). For genomic AI, the infrastructure question is not only “Can the data be found?” but also “Can the data be safely queried, correctly interpreted, and responsibly used for model development or evaluation?” Consent frameworks must also evolve alongside infrastructure. Original consent for clinical sequencing rarely anticipates secondary AI use, model training, or federated export. Dynamic or tiered consent architectures may be particularly relevant in small populations, where re-contact may be more feasible and where participant trust, community expectations, and downstream data reuse require careful governance. However, dynamic consent should not be presented as a universal solution; it introduces administrative burden, digital-access concerns, and potential consent fatigue (Dankar et al., 2020).

## 4 Discussion

### 4.1 From local validation to accountable deployment

Federated analysis provides one promising route. Instead of moving all genomic data into a central repository, models or summary computations can move to the data under controlled conditions (Montalvo et al., 2025; Zhou et al., 2024; Pati et al., 2024). This is particularly attractive for small biobanks that have legal, ethical, or cultural reasons to retain local control. Trusted research environments that bring the analysis to the data, such as Genomics England, the United Kingdom Biobank Research Analysis Platform, the All of us Researcher Workbench, and the European Genome-phenome Archive, illustrate this model, and the Five Safes framework (safe projects, people, settings, data, and outputs) offers a transferable governance vocabulary that small biobanks can adopt without large infrastructure investment (https://fivesafes.org/. https). However, federated learning still requires harmonised preprocessing, comparable phenotype definitions, robust security, protection against leakage from model updates, and transparent governance over who can run analyses and how outputs are released. The technical architecture and the governance architecture must be designed together.

Well-designed small genomic biobanks also provide a useful corrective to AI hype. The most clinically useful AI tools may not be the largest or most general models. In many genomic contexts, the immediate need is narrower: ancestry-aware variant frequency resources, splice-impact triage, phenotype-specific variant prioritisation, pharmacogenomic decision support, local PRS calibration, or identification of recurrent founder alleles. Smaller, task-specific models may be more interpretable, auditable, and deployable than opaque general-purpose systems. The key question is not whether AI can produce a prediction, but whether the prediction changes a clinical decision under defined conditions.

Explainability also needs to be understood differently in genomic medicine. It should not be reduced to a saliency map, a feature-importance ranking, or a retrospective explanation of why a model produced a particular score. For an AI output to be clinically useful, the explanation should point to a plausible and testable mechanism. This may include, for example, a predicted effect on protein function, splicing, gene regulation, transcript usage, tissue-specific expression, cellular pathway activity, inheritance pattern, therapeutic response, or measurable biomarker.

Small biobanks are particularly valuable in this context because they can link genomic variation to rich local clinical information, family history, founder effects, population-specific allele patterns, and opportunities for targeted follow-up. This makes them more than external validation cohorts. Realising this potential, however, depends on the depth and accuracy of the underlying clinical linkage and phenotyping. They can help determine whether an AI prediction reflects a real biological mechanism, a clinically meaningful association, or merely a statistical pattern learned from training data. In this sense, small biobanks can contribute directly to hypothesis generation by connecting model outputs to interpretable genotype-phenotype relationships that can be tested through segregation analysis, functional assays, longitudinal follow-up, or multi-omics validation.

A practical framework for AI-ready small biobanks should include five minimum elements. First, genomic data should be technically standardised, with explicit documentation of sequencing platform, coverage, variant-calling workflow, genome build, annotation version, and quality thresholds. Second, phenotype data should carry provenance, including source, date, coding system, certainty, and whether the label is clinician-curated, algorithmically inferred, or self-reported. Third, population descriptors should be used with precision, distinguishing genetic ancestry, geography, ethnicity, language, migration history, and social identity where relevant (Smith et al., 2025). Fourth, access should be tiered, with common aggregate information separated from rare-variant, individual-level, or highly linkable data. Fifth, every AI use case should require external validation, local calibration, uncertainty reporting, and post-deployment monitoring.

The governance implications are substantial. AI-driven genomic medicine will require model cards, data sheets, audit logs, clinical responsibility chains, procedures for updating models, and mechanisms for withdrawing or correcting outputs when evidence changes. These requirements align with broader trustworthy-AI principles such as fairness, universality, traceability, usability, robustness, and explainability (Lekadir et al., 2025). In genomics, however, these principles must be operationalised at the level of the variant, gene, phenotype, population, and clinical action. The regulatory architecture is also maturing. In the EU, many AI systems used for clinical diagnosis, particularly those associated with regulated medical devices, are likely to fall within high-risk obligations under the AI Act, including technical documentation, risk management, and post-market monitoring. In the United States, the FDA has issued guidance on predetermined change control plans for AI-enabled medical devices, reflecting the need to manage iterative model updates while maintaining safety and effectiveness. Small biobanks participating in model development or validation will need legal and governance infrastructure that most do not yet possess (Pati et al., 2024).

The field should therefore move beyond a binary distinction between large and small biobanks. Large biobanks are indispensable for discovery and model development. Small biobanks are indispensable for calibration, interpretability, privacy engineering, and real-world clinical translation. They reveal whether AI systems can survive contact with local population structure, imperfect phenotyping, rare alleles, health-system variation, and governance constraints. This argument should not be misread as suggesting that small biobanks can replace large-scale resources for model training or discovery. Their limitations are real: reduced statistical power, greater vulnerability to overfitting, relatedness structure, phenotype sparsity, local ascertainment bias, and limited representation of rare outcomes. It also follows that these contributions are not automatic. A small biobank does not mitigate bias or expose implementation failure simply by virtue of being small or locally situated. Whether it can do so depends on practical design choices, technical standardisation of sequencing and variant-calling pipelines, fit-for-purpose governance, and clinical linkage of sufficient depth and accuracy. A poorly standardised, weakly governed, or thinly phenotyped small collection may reproduce the very biases it is intended to detect or introduce new ones through small-sample instability and local ascertainment. Their distinctive value lies elsewhere. Small biobanks are best positioned as calibration environments, external validation cohorts, governance testbeds, and sources of population-specific biological insight (Table 1). In this role, their small size is not a defect to be concealed, but a design constraint that forces explicit attention to uncertainty, portability, and clinical context.

## 5 Conclusion

AI will not democratise genomic medicine simply by making prediction cheaper. It will democratise genomic medicine only if its predictions remain valid outside the datasets that made them possible. Small datasets are where that claim can be tested. The future of AI-powered genomic medicine will depend not only on bigger models, but on smaller, better-governed, locally accountable datasets that force those models to prove their clinical trustworthiness.

## Data Availability

The original contributions presented in the study are included in the article/supplementary material, further inquiries can be directed to the corresponding author.
